# The impact of hyperglycaemic crisis episodes on long-term outcomes for inpatients presenting with acute organ injury: A prospective, multicentre follow-up study

**DOI:** 10.3389/fendo.2022.1057089

**Published:** 2022-12-05

**Authors:** Zixiao Duan, Peiyang Song, Cheng Yang, Liling Deng, Youzhao Jiang, Fang Deng, Xiaoyan Jiang, Yan Chen, Gangyi Yang, Yu Ma, Wuquan Deng

**Affiliations:** ^1^ Department of Endocrinology, Chongqing Emergency Medical Center, Chongqing University Central Hospital, Chongqing, China; ^2^ Department of Endocrinology, The Second Affiliated Hospital of Chongqing Medical University, Chongqing, China; ^3^ Department of Endocrinology, Banan People’s Hospital of Chongqing, Chongqing, China; ^4^ Department of Endocrinology, Chongqing Southwest Hospital, Chongqing, China

**Keywords:** diabetes, hyperglycaemic crisis episode, acute organ injury, mortality, long-term prognosis

## Abstract

**Background:**

The long-term clinical outcome of poor prognosis in patients with diabetic hyperglycaemic crisis episodes (HCE) remains unknown, which may be related to acute organ injury (AOI) and its continuous damage after hospital discharge. This study aimed to observe the clinical differences and relevant risk factors in HCE with or without AOI.

**Methods:**

A total of 339 inpatients were divided into an AOI group (n=69) and a non-AOI group (n=270), and their differences and risk factors were explored. The differences in clinical outcomes and prediction models for evaluating the long-term adverse events after hospital discharge were established.

**Results:**

The mortality among cases complicated by AOI was significantly higher than that among patients without AOI [8 (11.59%) vs. 11 (4.07%), *Q* = 0.034] during hospitalization. After a 2-year follow-up, the mortality was also significantly higher in patients with concomitant AOI than in patients without AOI after hospital discharge during follow-up [13 (21.31%) vs. 15 (5.8%), *Q* < 0.001]. The long-term adverse events in patients with concomitant AOI were significantly higher than those in patients without AOI during follow-up [15 (24.59%) vs. 31 (11.97%), *Q* = 0.015]. Furthermore, Blood β-hydroxybutyric acid (*P* = 0.003), Cystatin C (*P* <0.001), serum potassium levels (*P* = 0.001) were significantly associated with long-term adverse events after hospital discharge.

**Conclusions:**

The long-term prognosis of HCE patients complicated with AOI was significantly worse than that of HCE patients without AOI. The laboratory indicators were closely correlated with AOI, and future studies should explore the improvement of clinical outcome in response to timely interventions.

## Introduction

Hyperglycaemic crisis episodes (HCE) refer to an acute diabetic complication that is most likely to cause short-term death. Diabetic ketoacidosis (DKA) and hyperosmolar hyperglycaemia syndrome have a morbidity of approximately 2.6% and 0.2% of diabetes, respectively. The morbidity of diabetes remains high, resulting in an annually increasing number of hospitalizations because of HCE, as well as high medical expenditure and severe social burden (1, 2). HCE patients are prone to acute organ injury (AOI), which involves myocardial infarction, acute kidney injury (AKI), and neurological injury. The risk of mortality will be significantly increased in HCE patients complicated with neurological changes (3, 4). In addition, the risk of organ failure and mortality after hospital discharge will also be considerably increased in HCE patients complicated with AOI (3, 5–7). The possible reason may be related to the continuous damage caused by AOI in the hospital and its extension to hospital discharge. Although HCE has been confirmed to increase the risk of dementia, malignancy, end-stage renal disease and cerebral ischaemic injury in previous studies (4, 8, 9), the impact of different AOIs on long-term outcomes in patients with HCE remains unknown. In this work, a prospective, multicentre follow-up observational study was conducted on inpatient HCE with or without AOI, and the adverse events after hospital discharge were investigated.

## Methods

### Patient recruitment and exclusion criteria

A total of 339 HCE patients were enrolled from 4 tertiary teaching hospitals between August 2017 and July 2020 in accordance with the principles of the Declaration of Helsinki (2001). This study was registered at chictr.org.cn (ChiCTR1800015981) and approved by the institutional review board of Chongqing University Central Hospital. The study was approved by the Committee for Research Ethics of Chongqing University Central Hospital (NO. 202262).Written informed consent was obtained from all participants.

Inpatients aged 20 or older diagnosed with HCE were enrolled in the study. All patients met the diagnostic criteria for diabetes and HCE recommended by the American Diabetes Association (ADA) in 2009 (10). Subjects with the following conditions or diseases were excluded: 1) pregnancy; 2) history of stroke/cognitive impairment, CKD, tumour, coronary heart disease/heart failure, chronic pancreatitis, or chronic hepatitis/liver failure; and 3) other major diseases, including systemic inflammatory response syndrome or advanced malignant diseases.

**Figure 1:**
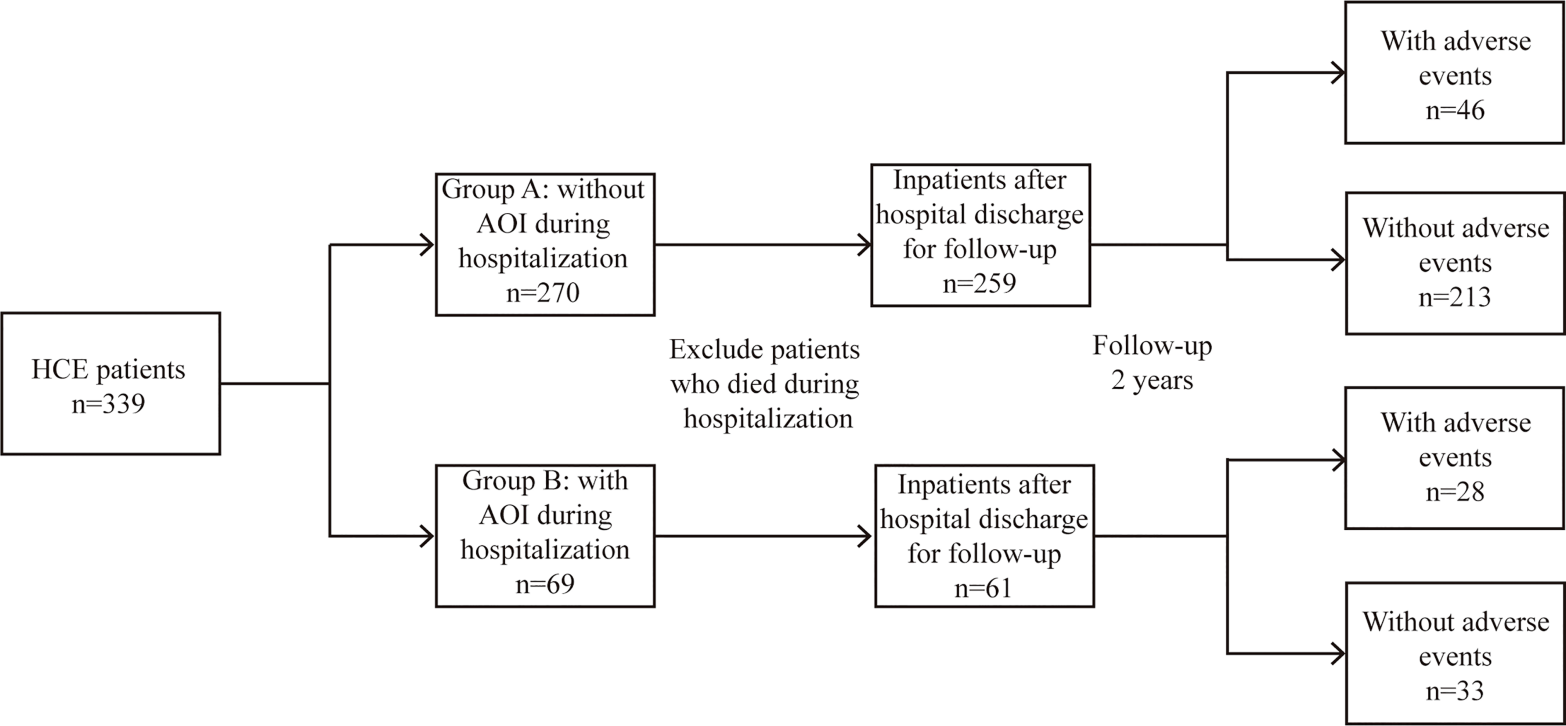
A flow chart describing the study design.

AOIs included the following: 1) acute stroke or brain dysfunction; 2) acute kidney injury; 3) acute cardiovascular accident/heart failure; 4) acute pancreatitis; and 5) acute liver dysfunction/failure. According to whether HCE was concomitant with AOI during admission, the patients were divided into an AOI group and a non-AOI group, and their differences and risk factors were explored. Long-term adverse events after hospital discharge were defined as follows: 1) stroke or cognitive impairment; 2) chronic kidney disease; 3) coronary heart disease or heart failure; 4) tumour; and 5) sudden death of unknown cause. The differences in clinical outcomes and a prediction model for evaluating the long-term adverse events after discharge were established **(** **Figure 1** **)**.

### Data collection

The baseline data of patients, including age, sex, body mass index (BMI), diabetes classification, predisposing factors, and AOI at admission and at discharge, were collected. BMI was calculated as the body weight/square of height [kg/m (2)]. The diabetes classification was determined by previous medical history.

### Biochemical assays

Blood glucose, blood sodium, blood potassium, C-reactive protein (CRP), procalcitonin (PCT), activated partial thromboplastin time (APTT), prothrombin activity (PTA), serum creatinine (SCR), cystatin C, creatine kinase, pH, HCO3-, base excess (BE), alanine aminotransferase (ALT), aspartate aminotransferase (AST), serum albumin, blood amylase, triglyceride (TG), total cholesterol (TC) and low density lipoprotein (LDL) were measured using an automatic biochemical analyser (AU5821, Beckman Coulter, USA). β-hydroxybutyrate was measured using a colorimetric enzymatic reaction (D-3-hydroxybutyrate kit; Ranbut, Randox Laboratories). White blood cells, neutrophils, lymphocytes, and platelets were determined by an automatic blood cell analyser (BC-6800, Mindray, Shenzhen, China). Troponin I was measured by chemiluminescence immunoassay using a DXI800 (Beckman Coulter, USA). Haemoglobin A1c was measured by high-performance liquid chromatography (BC-6800, Mindray, Shenzhen, China).

### Statistical methods

Normally distributed data are expressed as the mean ± standard deviation and compared using unifactor ANOVA and the TAMHANE test. Nonnormally distributed data are expressed as percentiles (P25, P75 and P50) and analysed using the rank-sum test. Count data are expressed as percentages (%) and were analysed using the chi-square test. All data were corrected for *P* value by false discovery rate method. Binary logistic regression analysis was performed after selecting the relevant factors using univariate logistic regression. By calculating the variance expansion factor, we confirm that there is no unacceptable collinearity in all regression models. The ROC curves were also plotted for the related factors. The SPSS 22.2 software was used for the clinical data analysis of enrolled patients. *P* value < 0.05 or *Q* value < 0.05 were considered statistically significant.

## Results

### Baseline clinical and laboratory characteristics

**Figure 2:**
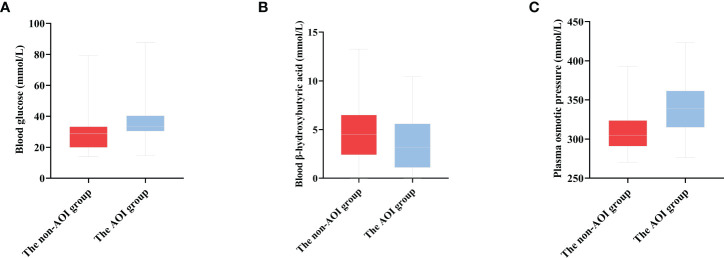
The distribution of blood glucose, blood ketone and plasma osmotic pressure in the two groups **(A)** The distribution of blood glucose in two groups [(30.43, 40.36) 33.30 vs. (19.90, 33.40) 28.70, *P* < 0.001]. **(B)** The distribution of blood β-hydroxybutyric acid in two groups [(1.10, 5.60) 3.18 vs. (2.40, 6.50) 4.50, *P* = 0.031]. **(C)** The distribution of plasma osmotic pressure in two groups [(290.83, 323.75) 304.20 vs. (315.0, 361.6) 339. 0, *P* < 0.001].

In this study, 339 HCE patients were enrolled. Among them, 69 cases were complicated by AOI, and 270 patients were free of AOI. At admission, the age, history of diabetes, blood glucose, plasma osmotic pressure, triglycerides, blood sodium, blood chloride, SCR, urea nitrogen, cystatin C, creatine kinase, troponin I, CRP, leukocytes, HCO3-, acute infection and deaths in the AOI group were significantly higher than those in the non-AOI group (*Q <*0.05), while blood ketone and serum albumin in the AOI group were unexpectedly significantly lower than those in the non-AOI group (*Q <*0.05). Type 2 diabetes was more common in the AOI group (*Q <*0.05). As shown in **Table 1** and **Figure 2**.

**Table 1:** Baseline characteristics of inpatients at admission or discharge.

Variables	AOI groupn=69	Non-AOI groupn=270	*Q* value
	n=69	n=270	
At admission			
Age	(44.50, 84.00) 65.00	(36.00, 66.00) 53.00	<0.001
Male	40/69 (57.97%)	159/270 (58.89%)	0.498
BMI	(19.60, 25.39) 22.50	(20.20, 25.10) 22.70	0.804
T2DM	66/69 (95.65%)	222/270 (82.22%)	0.005
History of diabetes, year	(0.75, 20) 8.5	(0.00, 10.00) 4.00	0.038
HbA1c (%)	(10.30, 13.90) 12.10	(10.20, 13.88) 11.85	0.791
Triglyceride, mmol/L	(1.60, 3.39) 2.37	(1.22, 2.99) 1.80	0.005
Blood Sodium, mmol/L	(133.50, 152.93) 140.40	(131.30, 139.70) 136.10	0.093
Blood Chlorine, mmol/L	(96.30, 115.63) 103.85	(96.03, 106.75) 100.50	0.024
SCR, umol/L	(92.15, 286.50) 144.20	(53.93, 113.77) 71.80	<0.001
Urea nitrogen, mmol/L	(8.96, 27.50) 13.89	(5.07, 12.70) 7.26	<0.001
Cystatin C, mg/L	(1.13, 2.80) 1.70	(0.62, 1.56) 0.85	<0.001
Lactate, mmol/L	(2.25, 4.13) 3.34	(1.83, 4.21) 2.57	0.066
Creatine kinase, U/L	(62.00, 365.00) 113.00	(54.25, 147.13) 73.00	0.040
Troponin I, ug/L	(0.00,0.11) 0.01 0.17 ± 0.61	(0.00, 0.02) 0.01 0.03 ± 0.09	0.005
Albumin, g/L	(30.10, 39.20) 36.30	(35.20, 43.93) 40.30	<0.001
CRP, mg/L	(4.61, 115.50) 22.45	(2.86, 35.58) 4.90	<0.001
White blood cells, 10 ^ 9/L	(9.62, 18.85) 12.70	(6.60, 14.40) 9.99	<0.001
pH	(7.25, 7.39) 7.31	(7.21, 7.37) 7.30	0.092
BE, mmol/L	(-13.73, -2.65) -7.25	(-17.53, -3.45) -8.90	0.308
HCO3-, mmol/L	(11.75, 21.48) 17.30	(7.80, 17.70) 14.40	0.006
Follow-up time, day	(326.50, 957.75) 604.50	(439.00, 1038.50) 728.00	0.298
Concurrent infection	51/69 (73.91%)	139/270 (51.48%)	0.001
**At discharge**			
Blood sodium, umol/L	(137.05, 143.4) 140.2	(136.4, 140.8) 138.9	0.093
Blood potassium, umol/L	(3.29, 4.15) 3.8	(3.55, 4.12) 3.84	0.322
Chlorine, umol/L	(101.45, 108.7) 105.1	(102.1, 107.2) 105.3	0.984
Urea nitrogen, umol/L	(4, 8.6) 5.4	(3.1, 6.83) 4.6	0.033
SCR, umol/L	(63.50, 168.05) 94.00	(49.00, 82.00) 62.50	<0.001
Cystatin C, mg/L	(1.03, 2.30) 1.50	(0.70, 1.44) 0.97	<0.001
Lactate, mmol/L	(1.72, 3.29) 2.44	(1.97, 3.06) 2.45	0.909
Creatine kinase	(48, 152) 63	(46, 132) 73	0.780
Troponin I, ug/L	(0.00, 0.07) 0.01	(0.00, 0.02) 0.00	0.017
Albumin, g/L	(28.35, 34.20) 31.20	(32.43, 38.10) 34.80	<0.001
CRP, mg/L	(6.15, 32.85) 14.15	(1.56, 31.50) 6.10	0.013
White blood cells,10 ^ 9/L	(3.69, 7.13) 4.63	(3.2, 5.32) 4.27	0.060
Blood platelets	(133.5, 329.5) 208	(152, 251.25) 200.5	0.312

AOI, Acute organ injury group; BMI, Body Mass Index; SCR, Serum creatinine; CRP, C-reactive protein; pH, Potential of hydrogen; BE, Base excess; Q < 0.05 (two-tailed) was considered statistically significant.

At the time of hospital discharge, the urea nitrogen, SCR, cystatin C, troponin I, and CRP levels in the AOI group were significantly higher than those in the non-AOI group (*Q <*0.05), and serum albumin was lower in the AOI group than in the non-AOI group (*Q <*0.05) (**Table 1**).

### Comparison of clinical outcomes during hospitalization and hospital discharge

**Figure 3:**
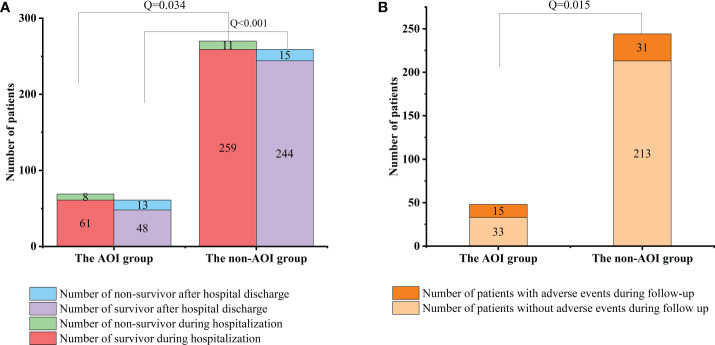
Comparison of mortality and follow-up between two groups during hospitalization and after discharge. **(A)** Comparison of mortality between the AOI group and non-AOI group during hospitalization and after discharge [8 (11.59%) vs. 11 (4.07%), *P* = 0.034; 13 (21.31%) vs. 15 (5.8%), *P* < 0.001]. **(B)** Comparison of follow-up between the AOI group and non-AOI group after discharge [15 (24.59%) vs. 31(11.97%), *P* = 0.015].

In this study, among the 69 HCE patients with AOI, 8 died during hospitalization, and among the 270 HCE patients without AOI, 11 died during hospitalization. The mortality among cases complicated by AOI was significantly higher than that among patients without AOI [8 (11.59%) vs. 11 (4.07%), *Q* =0.034] during hospitalization. Of the 61 HCE patients with concomitant AOI who recovered and were discharged from the hospital, 13 died, and 15 developed long-term adverse events during follow-up. Of the 259 HCE patients without AOI who recovered and were discharged from the hospital, 15 died, and 31 developed long-term adverse events during follow-up. The mortality was also significantly higher in patients with concomitant AOI than in patients without AOI after hospital discharge during follow-up [13 (21.31%) vs. 15 (5.8%), *Q <*0.001]. The long-term adverse events in patients with concomitant AOI were significantly higher than those in patients without AOI during follow-up [15 (24.59%) vs. 31 (11.97%), *Q* =0.015]. The short-term and long-term prognoses of cases complicated by AOI in the hospital were significantly worse than those of the group without AOI (**Figure 3**).

### Logistic regression analysis of factors related to AOI

ROC analysis showed that the sensitivity and specificity were 81.8% and 81.1% in the acute brain injury model (AUC 0.864, *P* <0.001), 85.7% and 81.2% in the acute cardiac dysfunction model (AUC 0.822, *P* =0.004), 67.7% and 76.7% in the AKI model (AUC 0.726, *P* <0.001), respectively (**Table S1**).

Significant factors were included in logistic regression analysis after univariate screening and collinearity analysis. Plasma osmotic pressure [aOR 1.032; 95% CI 1.014, 1.051; *P* <0.001], albumin [aOR 0.908; 95% CI 0.835, 0.988; P =0.026], and HCO3- [aOR 1.115; 95% CI 1.029, 1.209; *P* =0.008] were independent factors of acute brain dysfunction in HCE. Plasma osmotic pressure [aOR 1.020; 95% CI 1.008, 1.032; *P* =0.001] and SCR [aOR 1.003; 95% CI 1.001, 1.005; *P* =0.005] were related to AKI. SCR [aOR 1.004; 95% CI 1.002, 1.007; P =0.002] and troponin I [aOR 4.307; 95% CI 1.091, 17.009; *P* =0.037] were associated with acute cardiac dysfunction (**Table 2**).

**Table 2:** Logistic regression analysis of acute organ injury at hospital and adverse events after hospital discharge.

	β	*P*	OR	95%CI
**Acute stroke or brain dysfunction**				
Blood osmotic pressure	0.032	0.001	1.032	1.014~1.051
Albumin	-0.096	0.026	0.908	0.835~0.988
HCO3-	0.109	0.008	1.115	1.029~1.209
**AKI**				
Blood osmotic pressure	0.020	0.001	1.020	1.008~1.032
SCR	0.003	0.005	1.003	1.001~1.005
**Acute cardiovascular accident/heart failure**				
SCR	0.004	0.002	1.004	1.002~1.007
Troponin I	1.460	0.037	4.307	1.091~17.009
**Adverse events**				
Blood β-hydroxybutyrate acid	-0.396	0.003	0.673	0.516~0.878
Cystatin C*	0.820	< 0.001	2.271	1.437~3.589
Serum potassium ion*	-2.124	0.001	0.120	0.033~0.436
anion gap*	0.085	0.055	1.089	0.998~1.188

*Pre-discharge indicators.

AKI, Acute kidney injury; SCR, serum creatinine; P < 0.05 (two-tailed) was considered statistically significant.

### Logistic regression analysis for factors related to long-term adverse events

Blood β-hydroxybutyrate acid [aOR 0.673; 95% CI 0.516, 0.878; *P* =0.003], Cystatin C [aOR 2.271; 95% CI 1.437, 3.589; *P* <0.001], serum potassium levels [aOR 0.120; 95% CI 0.033, 0.436; *P* =0.001] were significantly associated with long-term adverse events after hospital discharge. In addition, anion gap [aOR 1.089; 95% CI 0.998, 1.188; *P* =0.055] was potentially associated with long-term adverse events (**Table 2**). The ROC analysis of this model showed a sensitivity of 84.6% and a specificity of 80.3% (AUC 0.887, *P* <0.001) (**Table S1**).

## Discussion

Our recent studies have confirmed that a high risk of mortality persisted in HCE patients with serious complications or AOI (11, 12). Age, sex, and comorbidity are regarded as important influencing factors for long-term AOI risk and mortality in HCE patients (10), which are commonly difficult to address with current clinical approaches. In addition, how to evaluate the acute and long-term AOI risk of HCE patients through laboratory indicators and how AOI affects the long-term prognosis of HCE patients remains unclear. This study demonstrated that the clinical outcomes of HCE patients with AOI were significantly worse than those of HCE patients without AOI in terms of both hospitalization and hospital discharge. Moreover, the analysis of influencing factors of AOI and long-term adverse events has laid a foundation for subsequent improved treatments. To the best of our knowledge, this model is the first in the Chinese population to predict long-term adverse events combined with AOI in HCE patients using laboratory indicators.

### Acute brain injury

Plasma osmotic pressure, plasma albumin and HCO3- levels were found to be important in predicting acute brain injury in patients with HCE. Reduced body volume caused by body fluid concentration may occur in a hypertonic state, and vasoconstriction may be mediated by Rho/Rho kinase signalling (13), further leading to insufficient blood supply to support important organs. Alternatively, exposure to hypertonic saline has been confirmed in current *in vitro* experiments to potentially cause brain cell death (13), and brain cells can be directly damaged by the hypertonic environment. In addition, the academic community widely recognized that the rapid reduction in osmotic pressure may exacerbate the risk of cerebral oedema in HCE patients. A previous study confirmed that the water content of the cerebral cortex is significantly increased in animals with reduced plasma osmotic pressure (14). Therefore, we believe that a persistent hyperosmolar state and rapid reduction in plasma osmotic pressure may lead to acute neuropsychiatric symptoms in HCE patients *via* the above mechanisms.

Albumin, a highly soluble protein, can act as an indicator to roughly estimate the nutritional level of patients. Despite its crucial role in maintaining the colloid osmotic pressure of human plasma, it also possesses complex roles, including the transport of various substances, immune regulation, stabilization of endothelial function, antioxidation, and inhibition of erythrocyte sedimentation (15). The serum albumin level has been suggested as a predictor of death, hospital stay and readmission time in acute patients. In this study, the albumin level was found to be an independent influencing factor of in-hospital acute brain dysfunction in HCE patients. Low albumin levels are partly involved in blood concentration, resulting in a further increase in plasma osmotic pressure and causing acute brain dysfunction *via* the aforementioned effects. In addition, hypoalbuminaemia may participate in acute ischaemic changes in the brain by affecting endothelial cell dysfunction and erythrocyte sedimentation (16).

In this study, HCO3-, an indicator of the degree of body acidosis, was found to be associated with acute brain dysfunction and cognitive decline in HCE patients. Hypocapnia can reduce cerebral blood flow and cerebral oxygenation (17), and insufficient cerebral blood supply and oxygen supply may be associated with vascular dementia or Alzheimer’s disease. In an acidic environment, the altered adhesion and morphology of microglia and astrocytes, along with the continuous decrease in scavenging activity of astrocyte Aβ (18), are similarly involved in acute brain injury. In addition, the unrelieved acidosis state during reperfusion may cause more serious damage to the brain (19).

### Acute kidney injury

Plasma osmotic pressure was found to be an independent factor affecting AKI in HCE patients. Similarly, in addition to acute brain injury, severe dehydration and blood concentration in the course of DKA were related to AKI. The kidney is highly vulnerable to plasma osmotic pressure. The reduced renal blood flow not only directly affects kidney function but also results in the reduction of renal oxygen supply and damage to renal cells. In addition, high osmotic pressure itself can also directly affect the kidney and induce cell apoptosis by affecting cell metabolism, nucleic acid stability and ion homeostasis (20), which is consistent with the fact that patients with isolated HHS or combined DKA had significantly more AOIs than patients with isolated DKA in this study. We speculated that this finding might be associated with the higher levels of plasma osmotic pressure. Therefore, this study concluded that higher plasma osmotic pressure indicates a higher morbidity of AKI for HCE patients. Therefore, active anti-infection and early active fluid rehydration therapy can play a positive role in protecting target organs from acute injury.

Creatinine is an endogenous marker for evaluating renal function, which is often influenced by infection, diet, and dehydration. Although its specificity and sensitivity are unsatisfactory, current guidelines suggest that serum creatinine remains an important marker for the diagnosis of AKI. Our study demonstrated a significant association between serum creatinine and AKI in patients with HCE. But because of the diagnosis of AKI is highly dependent on serum creatinine and the transient elevation of serum creatinine due to dehydration during HCE, we believe that there is not enough specificity in judging the risk of AKI by serum creatinine levels in the early stages of HCE. Similarly, due to severe dehydration, it is hard to judge the risk of AKI in HCE patients at an early stage based on their urine output. Current research indicates that Cystatin C, tissue inhibitor of metalloproteinases 2 (Timp-2) and insulin-like growth factor binding protein 7 (IGFBP7) are valuable markers for the diagnosis of AKI, but more studies are needed for HCE patients (21).

### Acute cardiac dysfunction

Some studies have found that elevated serum creatinine levels are associated with cardiac microvascular injury, but because of the strong association between serum creatinine and AKI, it may be that endothelial dysfunction and inflammation during AKI contribute to microvascular damage (22). Although patients with heart failure may have mild to moderate elevated SCR, it does not affect patient outcomes significantly (23). Therefore, it is believed that the increase of SCR in this condition is not caused by the renal function damage. Our study found that SCR was associated with the development of acute cardiac dysfunction in patients with HCE, but we do not believe that SCR is directly associated with acute cardiac dysfunction in patients with HCE because of the multiple reasons mentioned above. However, it has some value in predicting the cardiac dysfunction of the HCE patients.

Troponin I is an important marker of myocardial cell injury. At present, it is considered as an important indicator for the diagnosis of myocardial infarction, with high sensitivity and specificity. Our study found that the level of troponin I was related to the occurrence of acute cardiac dysfunction in HCE patients. Previous studies found that troponin I was elevated in patients with acute decompensated diabetes without clinical acute coronary syndrom (24). Our study also confirmed that there are still some patients with non-AOI group whose troponin I level is close to the upper limit of the laboratory reference value. At present, studies have found that in patients with acute myocardial infarction, hyperglycemia is related to larger infarct size and higher troponin I (25). Hyperglycemia may cause myocardial injury by mediating endothelial cell disorder and activating inflammatory mediators. It has been confirmed by *in vitro* experiments that 24-48 hours of hyperglycemia exposure can stimulate the production of inflammatory mediators in myocardial cells, leading to the elevation of markers of myocardial injury (25). Therefore, we believe that elevated troponin I is significantly related to the occurrence of acute cardiac dysfunction in HCE patients, but the elevated troponin I does not represent the occurrence of acute cardiac dysfunction in HCE patients. How to accurately determine the occurrence of acute cardiac dysfunction in HCE patients still needs further research.

### Long-term adverse events after hospital discharge

In this study, the levels of blood β-hydroxybutyric acid, serum potassium concentration, anion gap and cystatin C were found to be predictors for adverse events after hospital discharge in HCE patients.

Under the condition of long-term fasting or ineffective utilization of sugar for energy supply, fat decomposition will produce ketones, including acetylacetic acid, β-hydroxybutyrate and acetone, in which β-hydroxybutyrate accounts for the most. Blood-hydroxybutyrate levels are essential for the diagnosis of diabetic ketosis, and diabetic ketosis is closely associated with AKI. The increased ketone bodies were previously considered to exacerbate the body’s decompensation, resulting in an increased burden of target organs. Moreover, the increased level of blood β-hydroxybutyric acid was found to be a protective factor for HCE patients. Currently, several large-scale clinical studies have found that patients with ketosis have lower all-cause mortality during hyperglycaemic crises than those without ketosis (26, 27). Namely, HHS alone has a higher mortality than DKA alone or combined with HHS. Blood β-hydroxybutyrate can reduce renal ischemia and reperfusion injury by increasing the upstream regulator forkhead transcription factor O3 (FOXO3) and reducing caspase-1 and proinflammatory cytokines, thereby reducing cell death (28, 29). Moreover, serum β-hydroxybutyrate is also considered to be an antitumor agent (30). Exogenous ketone body intake can reduce atherosclerosis, improve heart failure and protect renal function by inhibiting inflammatory factors (31, 32) and improve cognitive function to some extent (33). Human proximal tubular cells can be directly damaged by high levels of blood β-hydroxybutyric acid (> 20 mmol/L) (34), while low concentrations of β-hydroxybutyric acid (<10 mmol/L) protect against ischaemic tissue injury. Nevertheless, blood ketones in HCE patients rarely exceed this level. Blood β-hydroxybutyrate may have no direct correlation with target organ injury in HCE patients but can reduce the risk of long-term adverse events in HCE patients. Therefore, appropriate ketogenesis can be beneficial to HCE patients. However, large-scale clinical studies are required to determine whether exogenous ketogenic supplementation is needed to improve the prognosis.

Serum potassium is involved in maintaining normal cell activity. Although malignant arrhythmia caused by severe hypokalaemia is significantly associated with the mortality of patients, studies on the relationship between hypokalaemia and the prognosis of HCE patients remain limited. Currently, studies have suggested that hypokalaemia is associated with cardiovascular events and renal adverse outcomes (35, 36), but the specific mechanism remains unclear. In this study, serum potassium concentration was found to be associated with the risk of adverse events after hospital discharge in HCE patients. Insignificant hypokalaemia is commonly associated with diuretics and poor nutritional status, and diuretics have been found to be related to long-term mortality and progression of renal disease (37). Poor nutritional status may lead to a heavy volume load and chronic inflammation by reducing plasma albumin levels, thereby resulting in higher all-cause mortality, cardiovascular events and infection-related mortality (38–40).

The anion gap is associated with coronary atherosclerotic heart disease and the risk of progression to end-stage renal disease in patients with CKD (41) and with the 30-day, 90-day, and 365-day all-cause mortality in patients with AKI (42). The anion gap mainly consists of lactic acid, phosphoric acid and ketone bodies. Lactic acid is often considered an indicator to predict the prognosis of patients with acute infection. Increased lactate levels usually indicate body hypoxia. Patients with diabetes are more likely to be infected than normal people, and aerobic glycolysis is essential for the normal immune function of cells. Conversely, lactic acid inhibits immunity in the local environment. Higher lactic acid may exacerbate the risk of reinfection or complications with diabetic foot ulcers in patients with HCE after discharge. The current study found a role for lactate in tumour occurrence and development (43), which may increase the risk of tumours in HCE patients after discharge. A previous study confirmed that compared with the normal population, DKA patients are prone to have a higher risk of developing Alzheimer’s disease after discharge (44). Considering the average age of HCE patients, acidosis should be corrected more actively than in the general population to improve the cognitive function prognosis of patients.

Cystatin C, a small protein molecule produced by nucleated cells, has been proven to be a highly sensitive biomarker of renal function. Compared with serum creatinine, cystatin C can serve as a stronger predictor for AKI. Several studies have confirmed that cystatin C can be used as a predictor of all-cause mortality in the elderly population, long-term mortality in patients with severe disease and mortality in patients with chronic kidney disease (23). Our study also proposed that cystatin C can be used as a predictor of diabetic foot ulcers (45). Cystatin C not only has a stronger ability than serum creatinine to predict mortality and the development of end-stage renal disease in diabetic patients (46, 47) but also has a strong correlation with the poor healing of diabetic foot ulcers (48) and the risk of cardiovascular disease, which seems to be associated with promoting atherosclerosis through multiple pathways (49). In this study, cystatin C was found to be not only a predictor of AKI but also an independent factor influencing adverse events after discharge in HCE patients. Although cystatin C seems to be correlated with multiple diseases, the direct involvement of cystatin C in the occurrence and development of the disease or its relationship with CKD remains unknown due to the sensitivity of cystatin C, which can be used as a diagnostic indicator of pre-CKD, and the lack of relevant clinical research.

Several limitations remain in this study. First, this work was a cross-sectional and observational study that did not include clinical intervention in HCE patients and potential improvements in clinical outcome. Second, the follow-up period was too short, and some patients could not provide detailed information, which may have led to the omission and ambiguity of a considerable portion of the positive outcomes. Therefore, we failed to conduct further analysis on the long-term outcomes and their influencing factors after discharge. Finally, we were unable to analyse DKA and HHS data due to the limited sample size and follow-up outcome.

## Conclusions

In summary, we established a prediction model to observe the impact of AOI on long-term outcomes in patients with HCE. The prognosis of HCE patients with concomitant AOI was found to be significantly worse than that of HCE patients without AOI. Further study should be performed to observe whether timely intervention for AOI can improve clinical outcomes.

## Data availability statement

The raw data supporting the conclusions of this article will be made available by the authors, without undue reservation.

## Ethics statement

The studies involving human participants were reviewed and approved by the Committee for Research Ethics of Chongqing University Central Hospital (NO. 202262). The patients/participants provided their written informed consent to participate in this study.

## Author contributions

ZD, PS and WD developed the protocol and were responsible for the study design. ZD, CY, LD, YJ and FD performed the systematic literature research and extracted data. XJ, YC and WD did data analysis and interpretation. ZD and PS wrote the first draft, and GY, YM and WD revised the article for important intellectual content. All authors contributed to the article and approved the submitted version.

## Funding

This study was supported by the Joint Medical Research Programs of Chongqing Science and Technology Bureau and Health Commission Foundation awarded to Dr. Wuquan Deng in 2023. The Fundamental Research Funds for the Central Universities (2021CDJYGRH-012) and the fund of Sichuan Provincial Western Psychiatric Association’s CSPC LEADING Scientific Research Project (Grant No. WL2021002) awarded to WD.

## Conflict of interest

The authors declare that the research was conducted in the absence of any commercial or financial relationships that could be construed as a potential conflict of interest.

## Publisher’s note

All claims expressed in this article are solely those of the authors and do not necessarily represent those of their affiliated organizations, or those of the publisher, the editors and the reviewers. Any product that may be evaluated in this article, or claim that may be made by its manufacturer, is not guaranteed or endorsed by the publisher.

## Supplementary material

Supplementary Table 1 Evaluation of target organ injury model in HCE patients. AKI, Acute kidney injury.
