# Supplementary material for: The impact of hyperglycaemic crisis episodes on long-term outcomes for inpatients presenting with acute organ injury: A prospective, multicentre follow-up study
Source: Front Endocrinol (Lausanne). 2022 Dec 5;13:1057089. doi: 10.3389/fendo.2022.1057089 (PMC9760800; doi:10.3389/fendo.2022.1057089)
Supplement: Supplementary Table 1 — Evaluation of target organ injury model in HCE patients. AKI, Acute kidney injury. [file Table_1.docx]

**Table S1. Evaluation of target organ injury model in HCE patients**

|  | AUC | *P* | sensitivity | specificity |
| --- | --- | --- | --- | --- |
| Acute stroke or brain dysfunction | 0.864 | <0.001 | 0.818 | 0.811 |
| Acute cardiac dysfunction | 0.822 | 0.004 | 0.857 | 0.812 |
| AKI | 0.726 | <0.001 | 0.677 | 0.767 |
| Poor prognosis | 0.887 | <0.001 | 0.846 | 0.803 |

AKI: Acute kidney injury
